# The Importance of Ordinal Information in Interpreting Number/Letter Line Data

**DOI:** 10.3389/fpsyg.2019.00692

**Published:** 2019-03-27

**Authors:** Christine Podwysocki, Robert A. Reeve, Jason D. Forte

**Affiliations:** Melbourne School of Psychological Sciences, University of Melbourne, Parkville, VIC, Australia

**Keywords:** ordered sequences, non-numerical order, ordinal information, number representation, number line, numerical estimation

## Abstract

The degree to which the ability to mark the location of numbers on a number-to-position (NP) task reflects a mental number line (MNL) representation, or a representation that supports ordered lists more generally, is yet to be resolved. Some argue that findings from linear equation modeling, often used to characterize NP task judgments, support the MNL hypothesis. Others claim that NP task judgments reflect strategic processes; while others suggest the MNL proposition could be extended to include ordered list processing more generally. Insofar as the latter two claims are supported, it would suggest a more nuanced account of the MNL hypothesis is required. To investigate these claims, 84 participants completed a NP and an alphabet-to-position task in which they marked the position of numbers/letters on a horizontal line. Of interest was whether: (1) similar judgment deviations from linearity occurred for number/letter stimuli; (2) left-to-right or right-to-left lines similarly, affected number/letter judgments; and (3) response times (RTs) differed as a function of number/letter stimuli and/or reverse/standard lines. While RTs were slower marking letter stimuli compared to number stimuli, they did not differ in the standard compared to the reverse number/letter lines. Furthermore, similar patterns of non-linear RTs were found marking stimuli on the number/letter lines, suggesting that similar strategic processes were at play. These findings suggest that a general mental representation may underlie ordered list processing and that a linear mental representation is not a unique feature of number *per se*. This is consistent with the hypothesis that number is supported by a representation that lends itself to processing ordered sequences in general.

## Introduction

Francis Galton (1880) was an early advocate of the position that space and number are related. More recently, it has been proposed that the brain represents numerical magnitude information on something akin to a mental number line (MNL; Dehaene, 2011). The MNL model suggests numbers are represented in ascending order from left-to-right in a “continuous, quantity-based, analogical format” (Zorzi et al., 2002, p. 138). Inferences about the nature of the MNL have been mostly based on data from a number-to-position (NP) task (see Siegler and Opfer, 2003), as well as studies on the spatial-numerical association of response codes (Dehaene et al., 1990, 1993), and spatial neglect (Zorzi et al., 2002).

In the NP task, participants mark the position of numerical values on a physical number line on which only the numerical end points are typically specified (e.g., ‘1’ and ‘100’). Findings show a close alignment (i.e., a linear algebraic function) between the marked positions of numerical values and their actual positions in older children and adults, while younger children typically overestimate small numbers and underestimate large numbers, and whose performance is best characterized by a logarithmic algebraic function. These findings are often used to suggest that the logarithmic/linear function that best fits NP task performance also characterizes the underlying form of the MNL. However, this interpretation should be treated with caution for at least two reasons. Firstly, it ignores the possibility that other processing factors affect, or may be responsible for, NP judgments. Secondly, it assumes that the NP/MNL relationship is unique to number. Clarifying these two possibilities may provide a more nuanced account of the relationship between number and space.

Evidence, albeit developmental evidence, suggests that performance on the NP task may not be a direct measure of numerical representations, and may depend on other processing factors (Barth and Paladino, 2011). NP estimation patterns instead may reflect an increased use of landmarks (the strategy of dividing lines into halves or quarters to create reference points to guide estimates) associated with a proportion judgment model, rather than a change in number representations *per se*. Barth and Paladino (2011) showed that children’s NP estimates were better fit by a proportion judgment model, rather than a “logarithmic-to-linear shift” account. This finding implies that performance on the NP task is not a direct measure of numerical representations because task strategies may influence NP task performance. While Barth and Paladino (2011) suggest NP task performance may be better described by a proportion power function rather than logarithmic/linear functions, they do not address whether NP task performance provides information about the representation of number *per se*.

It is clear that NP task manipulations may affect NP judgments. Cohen and Blanc-Goldhammer (2011; see also Cohen and Sarnecka, 2014), for example, used an “unbounded” NP task in which the number line represents a single unit distance from 0 to 1, and number estimations are made external to the bounds instead of within the bounds. On each trial, adult participants were presented with a target number (e.g., 12), and told to move the right boundary to estimate the target location. When participants completed a standard “bounded” NP task, estimation patterns closely followed a proportion judgment model, however, when they completed the unbounded NP task (which removes proportion judgment strategy opportunities), performance was best characterized by a linear function. This shows that task performance may reflect an increase in number line measurement skills, rather than a change in the underlying representation of number on the MNL (see also Huber et al., 2014). It also raises the issue of whether performance on a NP task reflects the way in which ordered lists, not just ordered number lists, are learned. Indeed, there is evidence that the pattern of mapping on the NP task may not be unique to number.

If number/letter line tasks, for example, produced the same mapping relationship to space, it would imply that linearity on the NP task may reflect a general mapping between spatial direction and list order. Several studies have investigated this possibility (Gevers et al., 2003, 2004; Berteletti et al., 2012; Hurst et al., 2014). For example, Hurst et al. (2014) gave children and adults an alphabet-to-position (AP) task, with the end points ‘A’ and ‘Z’. Children showed a logarithmic-to-linear shift with letters, and adults’ performance was characterized by a linear equation. While Hurst et al. (2014) were the first to show that numbers/letters spatial mapping abilities could be represented by linear/logarithmic functions, it is possible that performance on their task could be also represented by other algebraic functions (see comments on Barth and Paladino, 2011, above). Insofar as number/letter symbols display similar patterns on the spatial mapping task, it would argue against the claim that judgment patterns in the NP task are specific to number. Nevertheless, the issue of how best to compare similar/different number/letter judgment patterns, and ipso facto potential indices of different mental representations, remains an open question. In the present paper we address this issue by examining similarities/differences in non-linear deviations in NP and AP task performance.

In short, it is unclear whether NP judgment patterns are unique to number, or a general property of ordered lists that can be arranged spatially (e.g., the position of letters in an alphabet). To investigate this issue, we compared adults’ responses on NP and AP tasks, the aim of which was to determine whether the hypothetical mental representation of these two types of ordered lists differed. The research was designed to answer whether: (1) similar judgment deviations from linearity occurred for number/letter stimuli; (2) left-to-right or right-to-left lines similarly, affected number/letter judgments; and (3) response times (RTs) differed as a function of number/letter stimuli and/or reverse/standard lines. If patterns of spatial mapping and RT are similar for NP and AP tasks, it would suggest that NP task performance may reflect the way ordinal lists are processed, rather than anything specific about how numbers are represented. Such evidence would cast doubt on the value of drawing unique inferences about the underlying representation of number from spatial mapping tasks. Alternatively, if numbers/letters are found to have independent spatial mapping or RT patterns on NP and AP tasks, this would suggest that number symbols involve unique representations that can be studied using the NP task.

## Materials and Methods

### Participants

Eighty-four (*M* = 19.05 years, *SD* = 2.72 years; 30 males, 54 female) undergraduate students from an Australian university participated in the research for course credit (our sample size was determined by how many participants we were able to recuit via our first year research participation program, and is a similar size to the sample used by Berteletti et al., 2012). All participants had normal or corrected-to-normal vision. Written and informed consent was obtained by asking participants to read a plain language statement and sign a consent form. All procedures involved were approved by the University of Melbourne Human Ethics Advisory Group (HREC number 1441499).

### Apparatus

Stimuli were created on a Dell OptiPlex 9020 computer running Ubuntu with MATLAB software and Psychophysics Toolbox routines (Brainard, 1997; Pelli, 1997; Kleiner et al., 2007), and displayed on a 23 inch Dell E2314H LED monitor operating at a spatial resolution of 1,920 by 1,080 pixels at a refresh rate of 60 Hz.

### Stimuli

Participants completed a horizontal NP estimation task using integers between ‘1’ and ‘26’, and an alphabet-to-position (AP) task using letters between ‘A’ and ‘Z’ (with list positions that matched the numbers). In the NP task, the line endpoints were anchored with ‘1’ on the left and ‘26’on the right, or ‘26’ on the left and ‘1’ on the right (i.e., a reversed NP). Participants positioned a target number (2 to 25) along the line to indicate a location in space that corresponded to the number’s numerical position relative to the numerical endpoints. In the AP task, the lines were anchored with ‘A’ on the left and ‘Z’ on the right, or ‘Z’ on the left and ‘A’ on the right (i.e., a reversed AP). Participants positioned a target letter (B to Y) along the line to indicate a location in space that corresponded to the letter’s alphabetical position relative to the letter endpoints.

Participants were tested on half the possible items in the number/letter lists to minimize the total number of trials. Numbers/letters were chosen by selecting every second number and the corresponding list position letter between 2 and 25 (e.g., ‘3’ and ‘C’). Participants were randomly assigned to even number/letter or odd number/letter target symbol conditions. The even condition set used the twelve target numbers: 2, 4, 6, 8, 10, 12, 14, 16, 18, 20, 22, and 24, and the corresponding letters: B, D, F, H, J, L, N, P, R, T, V, and X. The odd condition participants were shown twelve target numbers: 3, 5, 7, 9, 11, 13, 15, 17, 19, 21, 23, and 25, and the corresponding target letters: C, E, G, I, K, M, O, Q, S, U, W, and Y. The target letter was displayed 8 cm above the middle of the horizontal line. The horizontal line was 15.5 cm long and 0.1 cm wide. The vertical marker used to indicate the target spatial position was 0.8 cm long and 0.1 cm wide. All stimuli were presented in black on a white background. Number/letter stimuli were presented in Arial font about 1 cm high.

### Procedure

Testing was conducted in a quiet testing space with participants seated about 60 cm in front of the computer monitor. Participants were presented with a series of horizontal lines on the screen, anchored by a number/letter on the left and right ends of the line. On each trial a number/letter appeared in the middle of the screen, above the line. Participants were instructed to move the computer mouse (i.e., vertical marker) left or right to the place where they thought the symbol should be on each line. Participants were asked to move the mouse marker and click when they thought they had positioned the symbol correctly.

Trials were randomized within a block of 48 trials (numbers/letters, left-to-right or right-to-left, 12 different targets), and participants completed nine blocks (i.e., 432 trials in total). Participants had as long as they wished on each trial to make their response. Trials were separated by 500 ms to reduce the potential for outlier bias in calculating RTs, participants with fewer than seven repeats for any condition were excluded from the analyses. Eighteen participants were excluded using this criterion, resulting in a final sample size of 66 participants, 79.57% of whom completed the testing.

### Analytic Approach

The overall aim of the analyses was to investigate similarities/differences in possible nonlinearities in the NP and AP data. As a first step though, we report the linear/logarithmic equation fits to our data. Specifically, we report the mean logarithmic and linear fit residuals for the NP and AP tasks to provide an overview of the data. To determine whether performance differed for the NP and AP tasks, we compared the spatial mapping RTs for number/letter symbols for the left-to-right version of the task, as well as right-to-left version of the task. The aim was to identify possible nonlinearities in the different tasks.

To more closely examine possible nonlinearities in these data, we plotted the average residuals from a linear fit for numbers/letters for both directions of the task. And to determine whether nonlinearities in the residuals depended on symbol type or task direction, we compared performance across numbers/letters and task direction. We also compared RT data for numbers/letters in both task directions to examine strategies used for NP/AP tasks.

To achieve the last three steps, we used a nested bootstrapping method to generate 95% confidence intervals. A distribution of group mean data was generated by calculating the mean of 10,000 samples of participants (with replacement) using resampled raw mapping data (with replacement) for each participant. To get a better idea of the underlying patterns in these data, we also fit polynomial curves to the data using the polyfit function in MATLAB.

## Results

Overall, the data are consistent with a linear mapping function. For each participant in each condition, we calculated the mean of the residuals from both a linear and a logarithmic mapping function as a proportion of the line length. In every case, the mean linear residual was lower than the mean logarithmic residual. The group average mean linear residual was 0.005, whereas the group average mean logarithmic residual was 0.064. This shows that a logarithmic model was not a good fit for any participant in any of the conditions, and that a linear model was a better general characterization of task performance.

### Mapping Between Symbols and Space

**FIGURE 1:**
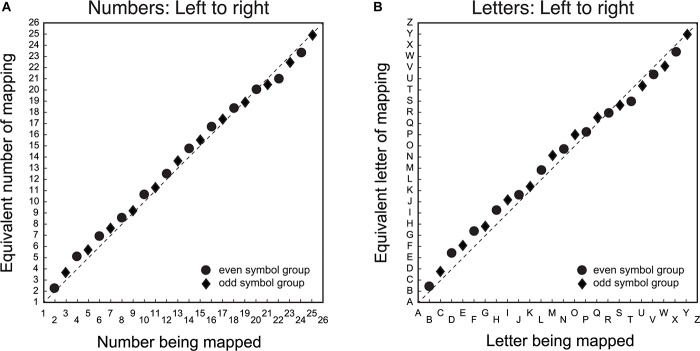
Group average symbol to line position matching for number/letter items. The *X*-axis is the symbol being matched, and the *Y*-axis is the equivalent symbol of the match. Panel **(A)** is the mapping data for number symbols from left-to-right and panel **(B)** is the corresponding data for the letter symbols from left-to-right. Circular data points are for the group that mapped even item position symbols. The diamond data points are for the group that mapped odd item position symbols. The dashed diagonal line is a veridical linear mapping between symbols and line position.

The first step was to determine whether there was a difference between number/letter symbols in the spatial mapping task. Figure 1 shows the group mean average symbol to line position matching for number (Figure 1A) and letter (Figure 1B) items. The solid symbols are the group averages of individual mapping data, which is the mean of 6 to 9 repetitions of each item. The data deviated minimally from the diagonal (the average residuals were typically less than 5% of the spatial distance between items).

It is evident that there is a general linear mapping between symbols and space that does not depend on symbol type. While the pattern of the mapping between symbol and space is similar for both numbers/letters, the systematic deviations from the diagonal suggest that these mapping functions both have non-linear components. Specifically, there was a tendency to mark the line further to the center of the veridical linear location of the symbol for items proximal to, but not immediately adjacent to, the end points. There was also a tendency for items in the middle of the list to be positioned toward the right of the veridical location.

Non-linear mappings of number to space have been reported previously (e.g., Siegler and Opfer, 2003). However, these nonlinearities are typically characterized as logarithmic or quasi-logarithmic patterns, where symbols are mapped toward the right of the linear prediction for all items. The present results differ from previously reported logarithmic patterns in two ways. Firstly, the nonlinearities are small. Secondly, the patterns of non-linear residuals, both above and below a veridical linear function, are not consistent with a mapping model based on a power or quasi-logarithmic function. It is possible the systematic nonlinearities in the spatial mapping function for numbers/letters may reflect a bias to either under- or over-estimate the position of number/letter items due to the properties of the task. Alternatively, the pattern of nonlinearities may reflect list order effects common to both numbers/letters when they are mapped to space.

### Mapping Between Symbols and Space With Reversed Mapping Direction

**FIGURE 2:**
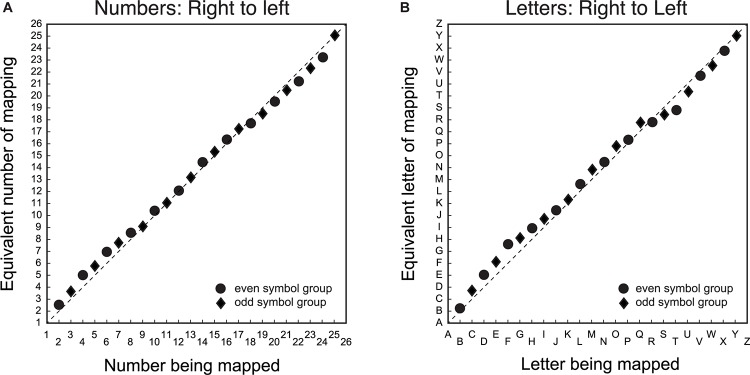
Group average symbol to line position matching for number/letter items when the left-to-right spatial direction was mismatched with symbol order. The *X*-axis is the symbol being matched, and the Y-axis is the equivalent symbol of the match. Panel **(A)** is the mapping data for number symbols from right-to-left and panel **(B)** is the corresponding data for the letter symbols from right-to-left. In the actual experiment, the left side of the line was ‘26’ or ‘Z’, and the right side was ‘1’ or ‘A’. Circular data points are for the group that mapped even item position symbols. The diamond data points are for the group that mapped odd item position symbols. The dashed diagonal line is a veridical linear mapping between symbols and line position.

The second step was to determine whether there was a difference between number/letter symbols in the spatial mapping task when the mapping direction was switched from left-to-right to right-to-left. Figure 2 displays the group mean average symbol to line position matching in the mapping task for number (Figure 2A) and letter (Figure 2B) items, where the spatial direction of the line was reversed by anchoring the left side of the line with ‘26’ or ‘Z’, and the right side with ‘1’ or ‘A’, respectively. The data are displayed in the item order rather than the spatial direction, because consistent patterns in the data depend on item position. As with Figure 1, the mapping between number symbols and line position deviates minimally from the diagonal, consistent with a linear mapping from number symbols to space.

Also similar to Figure 1, 2 shows systematic deviations above and below the diagonal that are qualitatively similar for both number/letter symbols. There is a tendency to map symbols onto the line further to the center of the veridical linear location of the symbol. Since the direction of the line was dissociated from item order, nonlinearities in the same direction for Figure 1, 2 reflect nonlinearities that are related to item order, whereas nonlinearities in the opposite direction are nonlinearities that depend on spatial direction.

### Group Average Residuals of Symbol to Line Mapping

**FIGURE 3:**
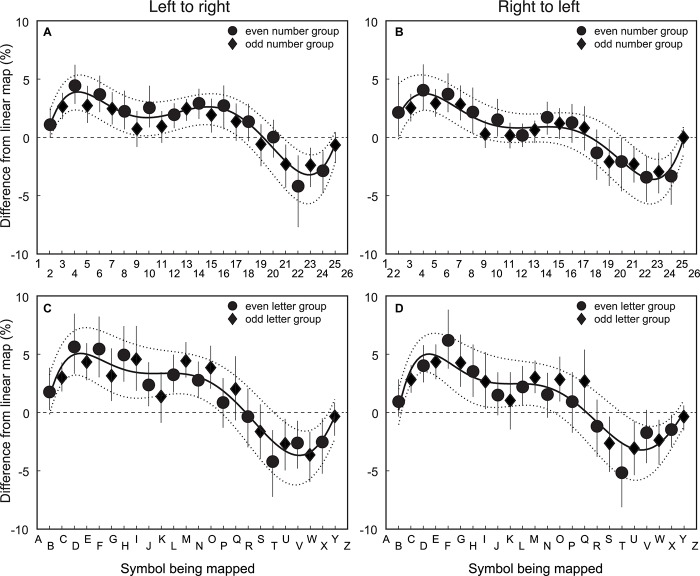
Group average residuals from veridical linear fit of symbol to line mapping for number/letter items for left-to-right and right-to-left spatial directions. The *X*-axis is the symbol being matched, and the *Y*-axis is the deviation from linear for the match. The top row **(A)** and **(B)** shows the data for numbers and the bottom row **(C)** and **(D)** shows the data for letters. The left column **(A)** and **(C)** shows the data for left-to-right item direction. The right column, **(B)** and **(D)** have the data for right-to-left item direction. Circular data points are for the group that mapped even item position symbols (*n* = 34). The diamond data points are for the group that mapped odd item position symbols (*n* = 32). Error bars are 95% CIs of the mean calculated using a bootstrapping procedure. The solid line is the best fitting sixth order polynomial to the group mean data. The dashed lines are the best fitting sixth order polynomial to the 95% CI estimates from the bootstrapped calculation.

The third step was to examine the group average residuals from the veridical location for the number/letter symbols in the spatial mapping task. Figure 3 shows that the residuals from the veridical linear are qualitatively similar for symbol type and mapping direction. Figure 3A,B show the data for numbers. Figure 3C,D show the data for letters. Figure 3A,C show the data for left-to-right item direction. Figure 3B,D show the data for right-to-left item direction. To determine if the residuals systematically deviate from a linear mapping function, a sixth order polynomial was fit to the 95% confidence intervals (CIs) using polyfit in MATLAB.

These data show that there is a tendency to map symbols that are a fourth or three-fourths of the way through the item list length toward the center item. The pattern appears to be systematic, given that all data display this trend regardless of symbol or mapping direction. Furthermore, given that the polynomial fit to the 95% CIs do not include 0, the non-linear pattern near the end points is unlikely to have occurred by chance.

The data in Figure 3 show a small systematic tendency for items toward the middle of the list to be positioned away from the start of the list. This is evident for both numbers/letters. The bias is larger for left-to-right ordered items, suggesting that this effect is strongest when the item order and spatial mapping direction are matched. The polynomial fit to the 95% CIs for the left-to-right mapping data provides support for the proposition that the bias is unlikely to have occurred by chance. The polynomial fit to the 95% CIs for the right-to-left letter data does not include 0, meaning the residuals represents a systematic bias. While the polynomial fit to 95% CIs for the right-to-left number data does not provide evidence of systematic bias, there is a trend in the same direction as the other conditions. The pattern of results suggests there are few differences in the nonlinearities across symbol type or mapping direction.

### Comparison of Mapping for Symbol Type and Mapping Direction

**FIGURE 4:**
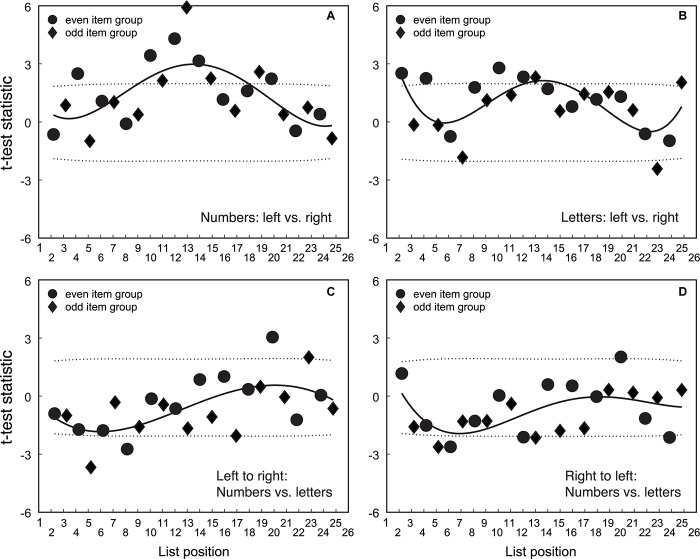
Comparison of mapping position across symbol type and mapping direction. Panel **(A)** shows data for the comparison of mapping direction for number symbols. Panel **(B)** shows data for the comparison of mapping direction for letter symbols. Panel **(C)** shows data for the comparison of number versus letter symbols for left-to-right mapping. Panel **(D)** shows data for the comparison of number versus letter symbols for right-to-left mapping. Solid symbols are *t*-scores computed for each comparison at each item position. The dashed line represents the 95% CIs of the distribution of *t*-scores obtained from randomly assigning data to groups. Solid symbols outside the dashed line indicate there is a significant difference between mapping position for conditions on a particular item. The solid line showing the trend in *t*-scores across items for each comparison is a third order polynomial. Circular data points are for the group that mapped even item position symbols. The diamond data points are for the group that mapped odd item position symbols.

The fourth step was to compare spatial mapping across number/letter symbols and mapping direction. Figure 4 displays the *t*-score for each item position. Figure 4A shows mapping direction for number symbols. Figure 4B shows mapping direction for letter symbols. Figure 4C shows symbol type for left-to-right mapping. Figure 4D shows symbol type for right-to-left mapping. To address how much the residual patterns depend on symbol type and the congruency between symbol item order and spatial direction, we performed a series of permutation *t*-tests for each item position, comparing mapping direction and symbol type. Comparisons of item direction were performed separately for each possible condition. In the permutation *t*-test, the 95% CI of the *t*-statistic for each data set was calculated using a bootstrapping procedure. The distribution of *t*-statistics was obtained by re-computing the *t*-value 10,000 times with random assignment of the data to each group. If the *t*-value of the data is outside the 95% CI for the bootstrap *t*-distribution, then we can infer that the data are different for the condition.

The trend in *t*-scores suggests that number mapping differs for left-to-right versus right-to-left direction for symbols in the middle of the list. There is a similar trend for letters, but the *t*-scores for letters are not as large as the number *t*-scores, or as consistently outside the 95% CIs. Although the *t*-statistics show that there are systematic differences in number mapping for left-to-right versus right-to-left, the magnitude of the bias is small. The large *t*-values for middle items are partly due to less variance (or greater consistency) of position data across individuals for middle number items. As such, there may be more precision in the estimates for numbers (and less so for letters) in the middle of the list.

In sum, the pattern of *t*-statistics suggests that left-to-right and right-to-left mapping is similar for numbers/letters. There is a small trend for larger differences between numbers/letters in position mapping for symbols at the top of the item list. However, there is little in the position mapping data that reveals differences in the spatial mapping of numbers/letters. Nevertheless, there are differences in the response times (RTs) taken to perform symbol to space mapping which may be clarified with further analysis.

### Response Times for Symbol Type and Mapping Direction

**FIGURE 5:**
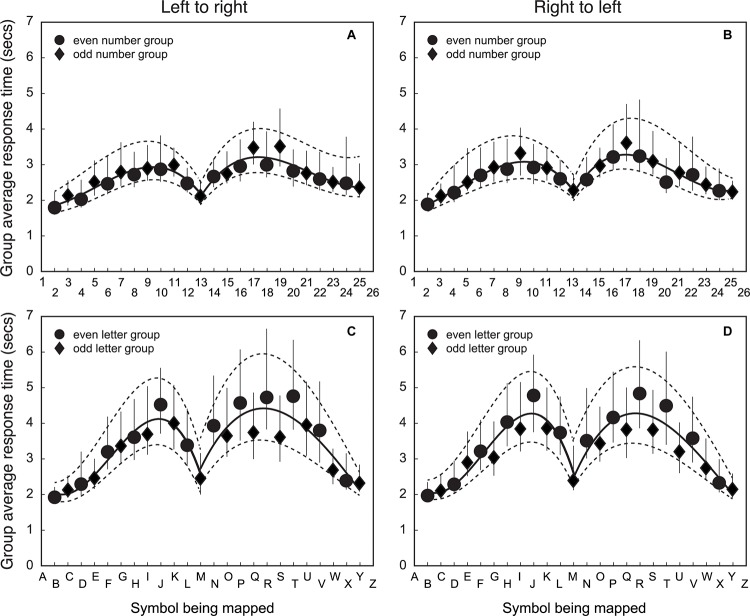
Group average RTs of matching a symbol to line position for number/letter items for left-to-right and right-to-left spatial directions. The *X*-axis is the symbol being matched, and the *Y*-axis is the group average response time for the match. The top row **(A)** and **(B)** shows the data for numbers and the bottom row **(C)** and **(D)** shows the results for letters. The left column **(A)** and **(C)** shows the data for left-to-right item direction. The right column, **(B)** and **(D)** have the data for right-to-left item direction. Circular data points are for the group that mapped even item position symbols (*n* = 34). The diamond data points are for the group that mapped odd item position symbols (*n* = 32). Error bars represent 95% CIs of the mean calculated using a bootstrapping procedure. The solid line is the best fitting third order polynomial to the group mean data for items in the upper and lower half of the symbol list (both fits include the 13th item). The dashed lines are the best fitting third order polynomial to the 95% CI estimates from the bootstrapped calculation (separately for the upper and lower halves of the data).

The fifth step was to compare RTs across number/letter symbols and mapping direction. Figure 5 shows group average RTs for matching symbols to line position. Figure 5A shows the RT to map numbers that are ordered left-to-right. Figure 5B shows the RT to map numbers that are ordered right-to-left. Figure 5C shows the RT to map letters that are ordered left-to-right. Figure 5D shows the RT to map letters that are ordered right-to-left.

The RT to map a symbol onto a line has a distinct pattern as a function of item position that does not depend on symbol type or mapping direction. Mapping is fastest for items at either end of the list, becoming slower for items further from the list end, with RTs being slowest for items about a third from the end. RTs then decrease for symbols in the middle of the list, with the 13th element of the list (‘13’ or ‘M’) being mapped almost as quickly as items at the ends of the list. Given that ‘13’ is half the list length of ‘26’ if counting from the top of the list, the RT data suggest that symbol list order is important for understanding the process of how symbols are mapped onto the line position.

The pattern of RTs does not seem to depend strongly on mapping order. The magnitude and pattern of change in RTs with item position in panel 5A is very similar to panel 5B. Panel 5C is also similar to panel 5D. The importance of the 13th item for numbers/letters suggests that item order is processed in a similar way regardless of the symbol type. However, the variability in letter RTs is much larger than that for number RTs, so a small effect would be less evident in these data.

Other patterns of RTs may provide information about how participants map number/letter symbols to spatial locations on a line. The RTs for ‘B’, ‘C’ and ‘D’ are similar to ‘2’, ‘3’ and ‘4’. Similarly, the speed for mapping ‘X’ and ‘Y’ is similar to ‘24’ and ‘25’. The RT variance is also smaller for these letters compared to those far from the end of the list. This is good evidence of a processing speed advantage when item positions are close to the beginning or end of the list. Such positions are also easier to calculate for letters. The letter items toward the middle of the list take longer to calculate, and this may be reflected in the longer RTs for letters toward the middle of the list compared to numbers.

Overall, the RT patterns indicate that the strategies used to map numbers to spatial position are similar to that used to map letters to spatial position. The mapping process requires finding the item position, converting that to a proportion of list length, and mapping that into visual space. Such a process does not require there to be a spatial arrangement of numbers along a number line. However, we now have evidence that numerical ability is necessary to complete a spatial mapping task with numbers/letters. This numerical requirement supports the idea that changes in the spatial mapping of numbers onto a line in the NP task may reflect increased familiarity with ordered lists, the speed an item position can be determined, and the ability to calculate a proportion from the item position and total list lengths.

## Discussion

We compared the pattern of responses for NP and AP tasks. Our aim was to examine whether mapping of number to space, as indexed by the pattern of responses in a NP task, is unique to number, or reflects the way in which ordered lists are spatially represented (i.e., the AP task). Of interest was whether: (1) similar judgment deviations from linearity occurred for number/letter stimuli; (2) left-to-right or right-to-left lines similarly, affected number/letter judgments; and (3) RTs differed as a function of number/letter stimuli and/or reverse/standard lines. Three findings are of note. Firstly, similar deviations from linearity were found for numbers/letters, suggesting that participants used similar methods for judging the position of symbols in the two tasks. Secondly, end point symbols did not affect performance. Thirdly, participants took longer to make AP compared to NP task judgments. These findings support the view that NP judgments may not be uniquely informative about the MNL because the pattern of performance was similar for number/letter symbols.

Our findings are consistent with those of other researchers who have argued that changes in NP judgment patterns reflect an increased use of proportion judgment strategies (Barth and Paladino, 2011), or increased list processing automaticity (Hurst et al., 2014). We also found systematic nonlinearities in both number/letter mapping patterns that may reflect a small bias for marking lines away from end points. These small linear deviations are not explained by combinations of linear and/or logarithmic mapping functions, which would produce only positive residuals. Our data do not support the view that mapping symbols onto a horizontal line depends uniquely on the numerical properties of the symbols associated with their location on a hypothetical MNL. Rather, our findings are consistent with a model in which the symbol position within an ordered list is used to map a symbol to a location on a horizontal line.

We found little evidence that mismatches in number/letter symbol position order, or task direction, alter the shape of the mapping function between symbols and space. Specifically, the patterns of non-linear residuals were similar regardless of symbol type and regardless of whether the spatial direction of the horizontal line was matched with numerical magnitude direction. We found a small trend for middle items to be estimated toward the right side of space (an effect that was more evident for numbers than letters), but the *t*-statistics for combinations of symbol type and direction congruence show that the patterns were similar across conditions. These findings are consistent with the claim that participants use a single method to map the position of number/letter symbols to the location along the horizontal line.

The RT data showed that responses were faster for items near the end of the list and close to the middle of the item sequence. This indicates participants were most efficient judging the position of symbols that reflected a fraction of a half. While letter judgments were slower than number judgments, when the item position of the letter is known, RTs for the comparable symbol sets was similar. For example, the relatively fast RTs of ‘13’ and ‘M’ suggest that it is more efficient to map symbols that map onto simple fractions. This is consistent with the view that mapping the line position of symbols requires the use of list length to calculate the position of an item as a proportion. This calculation does not depend on the line end points. In other words, our findings are consistent with a model that suggests that the ability to judge proportions is critical to performing the NP task.

We suggest that the linearity of judgments on NP tasks may reflect how ordered lists are learned (items in the beginning of number/letter sequences are learned before elements later in the list), rather than unique information about the MNL. This is because there is no numerical requirement for letters, which are items that are ordered without inherent magnitude (e.g., 1 + 2 is meaningful, whereas A + B is not), to be spaced linearly with a fixed interval between each item. Our findings support those of Hurst et al. (2014) and Berteletti et al. (2012), who show that older children and adults mark letters linearly on an AP task. They cast doubt on the value of making inferences about NP task performance as a measure of the underlying number representation. Specifically, the mapping process in NP judgments may not involve decoding numerical information from a MNL and suggests that NP judgments may reflect list order learning of numbers in formal education.

It is important to note that NP judgments may require “number” precision, irrespective of the elements being mapped. A participant must know where an item is in a list, be able to calculate the relative position in the list, and be able to match the relative proportionate location of items on the line. In other words, participants must be able to calculate relative proportions of items on the line. For numbers/letters, participants were faster and more accurate for items at the beginning and end of lines, and for items in the middle of the line, most likely because it was easier to calculate these proportions. If a participant diagnosed with dyscalculia (Butterworth et al., 2011) were to complete this task, for example, we would expect equally poor performance for the number/letter elements, since this participant might be incapable of calculating the relative proportions of the line, even if they knew the order of the letters. The fact that letter responses were slower overall suggests participants may be mentally mapping letters onto numbers to complete the task.

A possible limitation of our study is that we analyzed group data without regard to the possibility of individual differences. However, given that the standard errors were low, we think there were probably few systematic differences in mapping patterns. Nevertheless, future work should investigate whether individual differences are related to the ability to estimate item position and make proportion calculations. Furthermore, evidence that familiar letters near the end of the list were processed as quickly as numbers suggest that list familiarity may be important in shaping the methods participants use in line judgment tasks. Future work with artificial non-numerical symbols could reveal learning processes that are relevant to numerical learning more generally.

Overall, our findings support the claim that the NP task should not be taken as a unique measure of number along a hypothetical MNL. Our results are consistent with the idea that ordinality plays an important role in explaining the mapping function on the NP task, rather than the magnitude of number symbols being matched. It seems possible that instead of a number specific representation, number may be supported by a representation that is able to process ordered lists in general.

## Author Contributions

CP contributed to investigation, methodology, data collection, and editing the original draft. RR contributed to draft editing and supervision. JF prepared the methodology, coding, data analysis, draft editing, and supervision.

## Conflict of Interest Statement

The authors declare that the research was conducted in the absence of any commercial or financial relationships that could be construed as a potential conflict of interest.
